# Detection of flap malperfusion after microsurgical tissue reconstruction using hyperspectral imaging and machine learning

**DOI:** 10.1038/s41598-025-98874-4

**Published:** 2025-05-05

**Authors:** Marianne Maktabi, Benjamin Huber, Toni Pfeiffer, Torsten Schulz

**Affiliations:** 1 Innovation Center Computer Assisted Surgery (ICCAS), Faculty of Medicine, Leipzig University, 04103 Leipzig, Germany; 2 Hochschule Anhalt - University of Applied Sciences | Campus Köthen, Bernburger Str. 55, 06366 Köthen, Anhalt Germany; 3 Clinic for Orthopedics, Trauma Surgery, and Plastic Surgery, Leipzig University Clinic, Liebigstraße 20, 04103 Leipzig, Germany

**Keywords:** Free flap, Perfusion, Hyperspectral imaging (HSI), Machine learning, Plastic surgery, Medical research, Computational biology and bioinformatics, Machine learning

## Abstract

Hyperspectral imaging (HSI) has shown significant diagnostic potential for both intra- and postoperative perfusion assessment. The purpose of this study was to combine machine learning and neural networks with HSI to develop a method for detecting flap malperfusion after microsurgical tissue reconstruction. Data records were analysed to assess the occurrence of flap loss after microsurgical procedures. A total of 59 free flaps were recorded, ten of which demonstrated postoperative malperfusion, leading to necrosis. Several supervised classification algorithms were evaluated to differentiate impaired perfusion from healthy tissue via HSI recordings. The best flap classification performance was observed using a convolutional neural network using HSI based perfusion parameters within 72 h after surgery, with an area under the curve of 0.82 ± 0.05, a sensitivity of 70% ± 33%, a specificity of 76% ± 26%, and an F1 score of 68% ± 28%. HSI combined with artificial intelligence approaches in diagnostic tools could significantly improve the detection of postoperative malperfusion and potentially increase flap salvage rates.

## Introduction

Hyperspectral imaging (HSI) is a rapidly evolving technique capable of capturing spectral information about different types of lesions during surgery^1^. With a short measurement time of less than 5 s and its non-invasive nature, HSI facilitates intraoperative measurements and aids in surgical decision-making. Information about oxygen saturation or haemoglobin concentration at depths of up to 4 mm provides a valuable dataset for the development of machine learning algorithms in medical HSI^1^. The application of HSI in medicine has led to numerous attempts to diagnose various diseases^2^. With the introduction of automated machine-based algorithms, more accurate and efficient detection and classification of these diseases now seem possible^1^.

Machine learning approaches rely primarily on statistical models that identify and learn patterns to perform specific tasks. The combination of HSI and machine learning methods is mainly applied in classification or diagnostic tasks^1^. Some of the most common methods are decision trees (DT), random forests (RF), naive Bayes optimization and neural networks^3^. DT are popular machine learning algorithms used in both classification and regression tasks. They offer a structured and intuitive approach to predictions by recursively splitting data on the basis of different features. Notably, while DT have many strengths, they can also exhibit high variance and instability^4^. To address these issues, ensemble methods that combine multiple DT, such as RF or boosting, are commonly used to enhance performance and robustness^5^. Naive Bayes optimization refers to the process of tuning and improving the performance of naive Bayes classifiers. These classifiers are machine learning models based on Bayes’ theorem, which operates under the assumption of feature independence^6^. The most well-known machine learning models are neural networks, which are inspired by the structure and function of the human brain. These models are widely used for various tasks, including image recognition. Neural networks consist of interconnected units, known as artificial neurons or nodes, which are organized into multiple layers^3,7^.

Over the past decade, numerous articles have been published on studies that combine machine learning algorithms with HSI for disease detection. Diagnostic technologies have been developed for different types of cancer, such as stomach, brain, skin, colon, and oesophageal cancers^8–12^. However, the accuracy varies depending on the type of cancer, ranging from 63 to 94%^1^. Other classification systems have been described for skin lesions, white and red blood cells, and septicaemia^13–15^. Although several publications from German-speaking countries have discussed the use of HSI in reconstructive surgery, the application of artificial intelligence methods in this field has not yet been described^16–18^.

In reconstructive surgery, microsurgical free flap transfer has played a key role in clinical practice. Over recent decades, surgical techniques have improved significantly, making the reconstruction of complex tissue defects using free flaps a safe approach, with success rates steadily increasing to 95%^19^. Despite these advances, ischemia-related complications can still occur after pedicle or free tissue transplantation, leading to partial or complete flap loss. Most anastomotic failures occur within the first 24 h. Early reintervention in compromised flaps has been shown to significantly improve success rates. Following this, the frequency of failure decreases^20^. Therefore, the goal of postoperative care is to detect ischemia-related complications^21^. Typically, postoperative procedures rely on clinical assessment, such as visual perfusion control (e.g., a 3-s capillary refill within the first 72 h)^22^. Thus, HSI has been used in five different studies on free flap monitoring in free tissue reconstructive surgery. Different thresholds for physiological parameters, such as near-infrared perfusion, have been assumed to indicate ischaemia^16–18^. The purpose of this study was to evaluate the ability of different machine learning methods combined with HSI to predict outcomes after microsurgery and to determine its diagnostic value.

## Methods

### Dataset

A TIVITA Tissue Hyperspectral Camera System from Diaspective Vision GmbH (Am Salzhaff-Pepelow, Germany) was used to capture HSI data^23^. The system includes an illumination unit with two 120-W halogen lamps, an image spectrometer, and a complementary metal‒oxide semiconductor camera with a resolution of 640 × 480 megapixels for image recording. Owing to the interaction of light with the chromophores present in human tissue (e.g., melanin, haemoglobin), specific absorbance spectra are produced. By leveraging knowledge of absorbance spectra, calculations were derived, as described by Holmer et al.^2^. Different physiological parameters (tissue water index, saturation (StO_2_), near-infrared perfusion index (NPI), and haemoglobin index) can be calculated by using spectral channels from 500 to 1000 nm (5 nm resolution)^2^. For this study, we used wavelengths ranging from 540 to 1000 nm. StO_2_ and NPI led to valuable results in a flap monitoring study by Kohler et al.^16^. Therefore, in addition to spectral data, we used StO_2_ and NPI data. StO_2_ measures microcirculation in tissue up to a depth of 1 mm, whereas near-infrared light penetrates deeper tissue layers up to 4 mm.

**Fig. 1:**
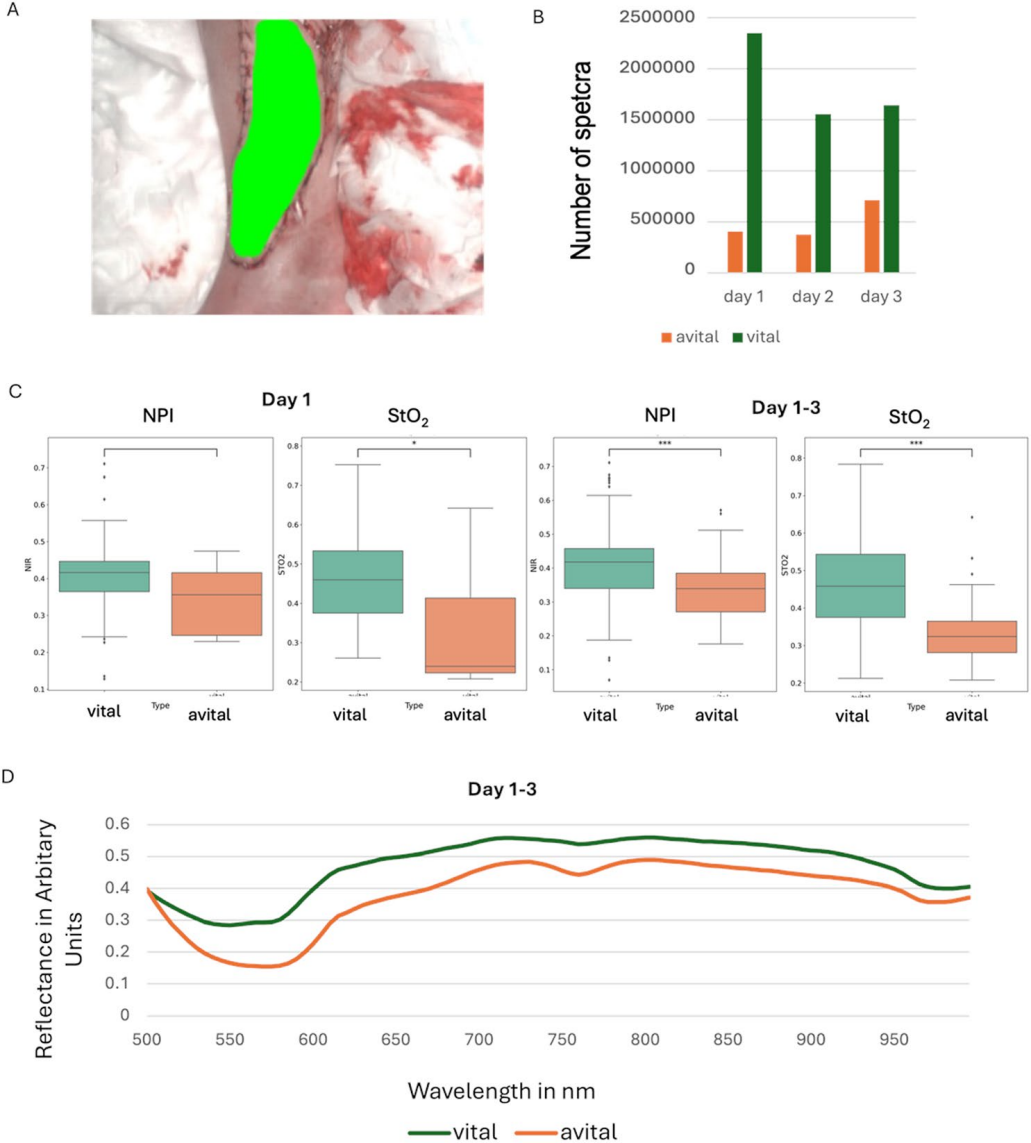
Distribution of the dataset on postoperative days, tissue vitality, and pixel count. **A** An example of a flap annotation. **B** Number of spectra for each day. **C** Differences in NIP and StO_2_ values between vital and avital tissue (*** p < 0.001, *p < 0.05). **D** Reflectance variations in vital and avital flaps within the first three days.

To analyse postoperative tissue viability, we collected HSI data from patients at different time points, categorising each case as either vital or avital (**Error! Reference source not found.**1). To ensure accurate classification, we manually annotated the hyperspectral images, segmenting regions of vital and avital tissue. These annotations, performed by an experienced surgeon, served as essential reference data for training and validating our machine learning models (Fig. 1A). The dataset includes data from 59 patients resulting in a total of 322 datasets. Among these patients, 11 patients were classified as avital, and 48 patients were classified as vital. Most data were recorded on the first postoperative day, with a gradual decrease in sample size over the following days. The dataset was imbalanced, as vital flaps accounted for the majority of cases, while avital flaps were less frequent, presenting a challenge for model training and validation (Fig. 1B). Regarding to the NPI and StO₂ values, our analysis revealed that both parameters were significantly higher in vital flaps, with statistical significance at p < 0.001 and p < 0.05. Despite the overall distinction between groups, some overlap in parameter values was observed (Fig. 1C). Further spectral reflectance variations were examined over the first three postoperative days, tracking changes in both vital and avital flaps. The reflectance profiles of vital flaps remained relatively stable, whereas avital flaps exhibited deviations, reflecting progressive changes in tissue characteristics. By collecting spectral data over multiple days, we were able to capture temporal dynamics that could enhance model performance in distinguishing between viable and non-viable tissue (Fig. 1D).

### Data preprocessing

This section provides a detailed overview of the data processing steps. Only the annotated regions were considered in this study. As already mentioned, the dataset was imbalanced, with most pixels classified as vital and fewer than a quarter classified as average (Fig. 1C). In this work, we used the spectral data from 540 to 1000 nm and the physiological parameters StO_2_ and NPI as input data. Furthermore, three-dimensional data and one-dimensional data were used. Oversampling was applied using the synthetic minority oversampling technique (SMOTE) for one-dimensional data^24^. This technique was only applied to the training set. Class weights were used when working with three-dimensional data^25^. Three-dimensional data were extracted by using a patch size of 3 × 3 pixel. When spectral data were used as model inputs, the standard normal variance was calculated, and smoothing was applied along the spectral axis using the Savitzky‒Golay filter to reduce the effect of noise. To smooth the data in the spatial domain, median filters with a patch size of 11 × 11 pixel were used. To smooth the perfusion parameters, a Gaussian filter with a size of 11 × 11 was applied. If perfusion parameters were including, no further preprocessing was performed.

### Model selection

Selecting an appropriate model is a critical step in developing classification algorithms. We evaluated various models for classifying tissue vitality, considering several factors: spectral axis complexity (using either two dimensions or 92 dimensions), interpretability, computational effort, and the model’s adaptability to future data or changing conditions. We tested several pixelwise classifiers, including logistic regression (LR), multilayer perceptron (MP), support vector machine (SVM) and RF for the one-dimensional physiological and one-dimensional spectral data. For the three-dimensional physiological parameters and spectral data a well-known 3D convolutional neural network (CNN) was used described by Tkachenkoet and colleagues^25^. This remote-sensing based architecture consists of 3D convolution layers followed by 1D convolution layers (Fig. S1). For the physiological parameters a reshaping was done, and the 3D convolutional layers weren’t used. The models were trained with the adaptive moment estimation (Adam) optimizer (with β1 = 0.9 and β2 = 0.99) and a batch size of 100. Here, 8396 trainable parameters were used for spectral data and 4096 trainable parameters for perfusion data. The models were trained using an Nvidia GeForce RTX 2080 Ti. The training was performed by using early stopping with the F1 score.

To enhance model generalizability, hyperparameter optimization, early stopping, and leave-one-patient-out cross-validation (LOPOCV) were employed^25^. To test each method, 59 models were trained. For the standard machine learning models, the dataset was divided into a training set (N = 58 patients) and a testing set (N = 1 patient), and for the CNN, data from four patients were used for validation, while those from 54 patients were used for training. Hyperparameter optimization was performed for the RF, SVM, MP and CNN using Bayesian optimization with a small representative dataset (1% of the whole dataset). On the basis of the findings of Kohler et al.^16^, a DT based on a threshold of 40% was also tested. If the NPI or StO_2_ value was less than or equal to 40%, the pixel was classified as avital.

### Metrics and statistical tests

To compare different methods, general standards are needed. In this work, accuracy, sensitivity, specificity, the weighted F1 score, receiver operating characteristic (ROC) score, and the area under the curve (AUC) score were used. Specificity measures how well the model identifies true negative results and is calculated by dividing the number of correct negative predictions by the total number of actual negative cases^25^. Sensitivity, also known as recall, measures how well the model identifies true positive results by dividing the number of correct positives by the total number of actual positive cases^25^. The AUC score was calculated to provide a threshold-independent performance measure^26^. Statistical significance was evaluated using a t test.

## Results

### Classification

**Fig. 2:**
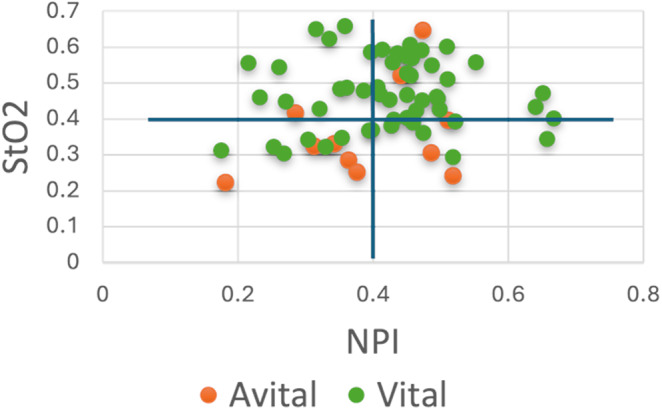
Patient-wise calculation of NPI and StO_2_ for day 1–3 (avital; orange; vital: green).

Five trained models were tested, including the RF, SVM, MP, LR, and CNN, as well as an untrained DT. The models were evaluated using both the physiological parameters NIP and StO_2_, as well as spectral data. Image data were available on the first postoperative day and within 72 h after the surgical procedure. The spectral data consisted of 92 wavelengths. The F1-score ranged between 79.51 and 100 (Table S1). The untrained DT was based on the threshold approach demonstrated in Fig. 2. A StO₂ and NPI value below 40% were classified as avital. Figure 2 illustrates the threshold-based approach using all acquired data from day one till three, where pixels from approximately two patients were misclassified as vital.

The highest diagnostic accuracy was demonstrated by the CNN and the untrained DT (Table 1).

**Table 1:** Calculated metrics using physiological parameters (pp) and spectral data (sd).

Method	Input data	Days	Sensitivity	Specificity	F1-score (weighted)	Area under the curve
Convolutional neural network	pp	1 day	0.66 ± 0.36	0.69 ± 0.28	0.60 ± 0.36	0.92 ± 0.08
Convolutional neural network	pp	1–3 days	**0**.**70 ± 0**.**33**	**0**.**76 ± 0**.**26**	**0**.**68 ± 0**.**28**	**0**.**82 ± 0**.**05**
Decision tree	pp	1 day	0.49 ± 0.39	0.00 ± 0.00	0.09 ± 0.25	–
Decision tree	pp	1–3 days	0.61 ± 0.30	0.07 ± 0.12	0.11 ± 0.27	–
Convolutional neural network	sd	1 day	0.57 ± 0.35	0.80 ± 0.30	0.52 ± 0.29	0.83 ± 0.10
Convolutional neural network	sd	1–3 days	0.61 ± 0.33	0.84 ± 0.21	0.62 ± 0.26	0.86 ± 0.12

The machine learning program with the highest diagnostic precision is in bold..

In detail, the highest sensitivity was achieved using the CNN with physiological parameters from all postoperative day reaching 70%. The CNN using spectral data from all postoperative days demonstrated the highest specificity with 84%. This was indicating a stronger ability to correctly classify vital tissue. When only a single day’s physiological data was used, the CNN achieved a specificity of 69%. The specificity was improving to 76% when multiple days were included. The highest weighted F1 score was reached with 68% for the CNN using physiological parameters including data from all postoperative day. However, the CNN trained with spectral data over three days exhibited a comparable F1 score of 62%. On the other side, the sensitivity was lower. The AUC score was highest for the CNN using physiological data from a single day with 92%. The spectral data-based CNN achieved an AUC of 86%.

The highest sensitivity combined with the highest specificity was demonstrated by the CNN when considering physiological parameters. The dataset included the first three postoperative days. The AUC for this model was 0.82, with an F1 score of 0.68. The DT approach, although sensitive with 61%, showed a dramatically decreased specificity with 12%.

### Visualization

**Fig. 3:**
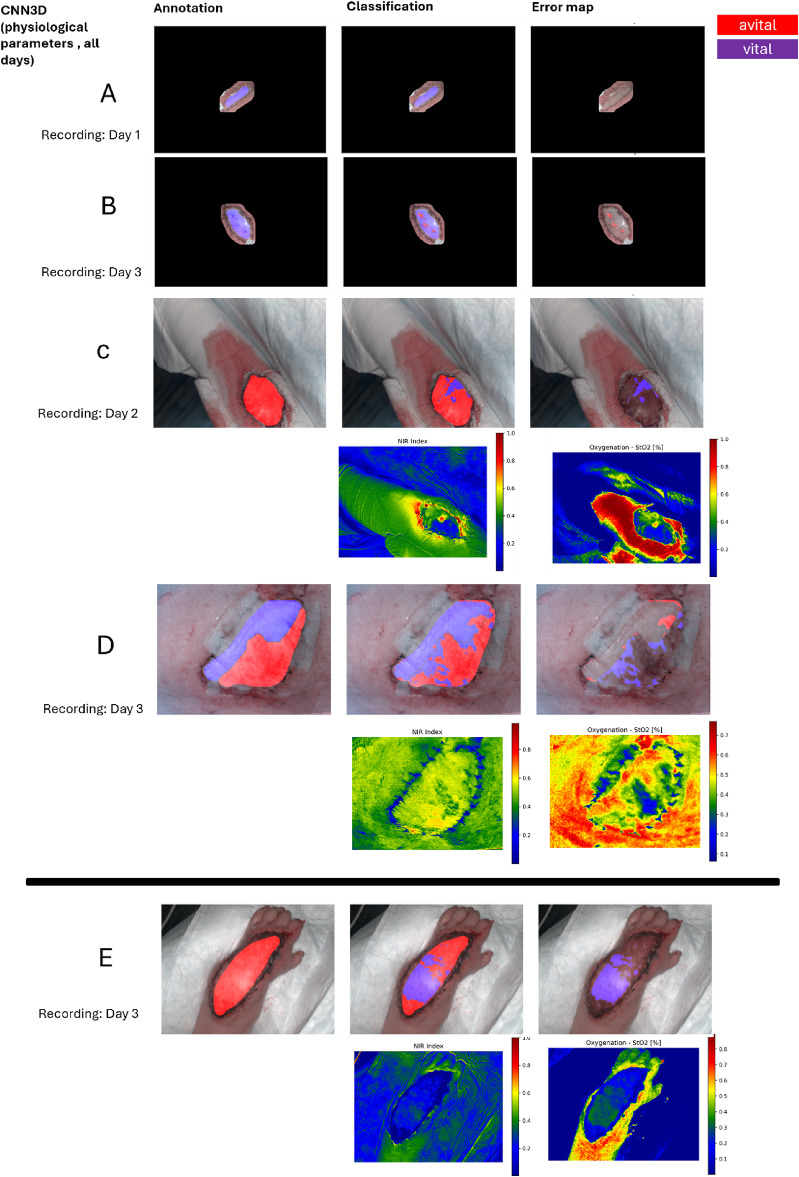
Annotation, prediction results, and error maps by the convolutional neural networks (physiological parameters, all days). Of patients **A**–**E**. Patient **A**–**D** are correctly classified. Patient **E** is misclassified. The physiological parameters of Patient **C**, **D** and **E** are shown.

Prediction images were generated to verify and visualize the classification results of our CNN for flap perfusion assessment. In the majority of all cases, all the pixels were correctly classified, highlighting the model’s ability to distinguish between vital and avital tissue (Fig. 3A–D). However, there were some predictions where certain pixels were misclassified, particularly in avital flaps. These errors occurred despite the fact that these examples displayed perfusion behaviours, which are expected for avital flaps (Fig. 3E). These mistakes suggest that additional information may be necessary to further refine the model’s sensitivity. In total, 43 patients were correctly classified, 11 patients showed partially misclassifications, and in 5 patients, misclassification of the entire flap was observed by using the CNN with physiological parameters from all postoperative days.

## Discussion

The results of our study demonstrate the potential of HSI combined with machine learning for the detection of flap malperfusion following microsurgical tissue reconstruction. Our CNN achieved a good performance using perfusion parameter with an F1-score of up to 68%. These findings support the hypothesis that deep learning models can successfully use HSI data to distinguish between vital and avital tissue. Our findings align with previous studies that have explored the application of HSI in microsurgical free flap monitoring. Kohler et al. demonstrated that HSI could detect ischemic complications earlier than clinical assessment^16^. Similarly, Thiem et colleagues reported that HSI outperformed conventional methods for perfusion monitoring^17^. However, these studies primarily relied on predefined perfusion thresholds rather than machine learning-based algorhytms. Our trial was able to expand these findings by demonstrating that deep learning models can further automate the process of tissue viability assessment.

In this study, two different types of input data (physiological parameters and spectral data) were used to classify flaps as vital or avital. Both input types were derived from HSI data, with the physiological data being calculated using specific ratios of the spectral data. One of the key findings was the superior performance of the CNN compared to other machine learning algorhytms. These results align with the current literature^27^. For example, the use of CNNs in dental disease classification achieved an accuracy of 98.6%^27^. Moreover CCN demonstrated better performances than traditional machine learning methods such as SVM^27^.

Further CNN demonstrated in our trial an increased performance when trained on physiological parameters compared to spectral data. Although full spectral information should provide additional discriminative information. This finding indicates that the information provided by physiological parameters is crucial for model decision-making. Schulz et al.^18^ noted that the physiological parameters StO_2_ and NPI differ between avital and vital flaps. A key advantage of using perfusion parameters is that they are calculated from absorbance data ratios, making them less susceptible to noise than are raw absorbance values in the spectral data. Another major advantage is that the number of features required to train the models is significantly reduced (two instead of 100 values), allowing for faster calculations and the ability to test more complex models and methods. In addition, practical aspects for using only a few wavelengths should be mentioned here. If only a few wavelengths are necessary for correct clinical assessment, a technological implementation based on multispectral approaches is possible. An advantage of multispectral approaches is their capability for real-time processing.

The DT model, which applied a simple threshold-based classification approach using physiological parameters, also performed well in terms of sensitivity (61%). However, the specificity was to low (12%). In contrast, the CNN maintained a better balance between sensitivity and specificity. In fact, the CNN seem to demonstrate a higher potential for clinical implementation minimizing false positives and false negatives results. Moreover, the CNN is able to process spatial and spectral HSI data in comparison to a DT model. The difference between day one and day three post-surgery suggests that increasing the input data for both vital and vital cases positively impact performance. Incorporating multi-day data (day 1–3) compared to single-day data (day 1) improved specificity but did not necessarily enhance sensitivity in regard to CNN and DT models. This suggests that while temporal information can help refine classifications, early postoperative perfusion patterns already provide strong indications of tissue viability in terms of the sensitivity. Physiological parameters yielded good results for the CNN. However, with increased computational power and time, neural networks could be effectively applied to the absorption spectra.

The neural network performed not automatically better when all the spectral data were used. Although, it is the most computationally intensive method.^28^ In contrast, the DT approach, which is computationally light, yielded acceptable sensitivities when only physiological parameters were used. The network performed better with absorption spectra when more data (HSI cubes from three days post-surgery) were used. The visualization confirmed that, while the CNN performs well in most cases (43 of 59 patients), some misclassifications remain (16 of 59 patients), particularly in flaps with atypical perfusion behaviour. Additionally, techniques such as data augmentation or transfer learning could improve model performance. These approaches should be tested in future studies.

Our study has several limitations that must be addressed. First, the dataset was imbalanced, with a higher proportion of vital flaps compared to avital cases. While we applied oversampling techniques such as SMOTE to address this issue, class imbalance may still have influenced model performance. In our study, HSI cubes were recorded at a distance of 50 cm. The recorded tissue showed only slight deviation from a flat surface. Nevertheless, slight deviations are expected to influence the spectral data. In a study by Studier-Fischer et al., the influences of different angles of view were investigated, revealing that the angle of view had only a small influence on the results^29^. However, environmental factors such as lighting conditions and tissue angle during image acquisition could introduce variability in spectral measurements. Although we standardized data collection as much as possible, small variations may still have impacted the results. Third, although our data collective showed that the CNN demonstrated sufficient diagnostic power, future studies should continue to test the external validity of both the CNN and the DT model using both physiological and spectral data to ensure external validity.

Physiological parameters significantly influence decision-making, as has already been demonstrated in various publications^16–18^. In future studies, it would be interesting to explore whether these two parameters alone are sufficient for accurate classification. This could reduce the computational load and enable the use of real-time multispectral camera systems. The difference in results between the postoperative days is not statistically significant and is likely due to the size of the underlying dataset. Future studies should investigate this in more detail to determine whether the improved results on day three are due to the dataset size.

## Conclusion

This study demonstrated that the classification of flaps as avital or vital using hyperspectral data combined with standard machine learning and deep neural networks is feasible. While threshold-based approaches remain a useful tool, CNN offer higher accuracy and a better balance between sensitivity and specificity. Using the full spectral data did not automatically result in significantly better performance than using only the physiological parameters. Because of imbalanced data sets, future studies should further explore the external validity of our findings.

## Supplementary Information

Supplementary Information 1.

Supplementary Information 2.

## Data Availability

The data supporting the findings of this study are available upon request from the corresponding author. However, they are not publicly available due to privacy or ethical restrictions.
