# Supplementary material for: Detection of flap malperfusion after microsurgical tissue reconstruction using hyperspectral imaging and machine learning
Source: Sci Rep. 2025 May 5;15:15637. doi: 10.1038/s41598-025-98874-4 (PMC12052805; doi:10.1038/s41598-025-98874-4)
Supplement: Supplementary file 1 — Supplementary Information 1. [file 41598_2025_98874_MOESM1_ESM.pdf]

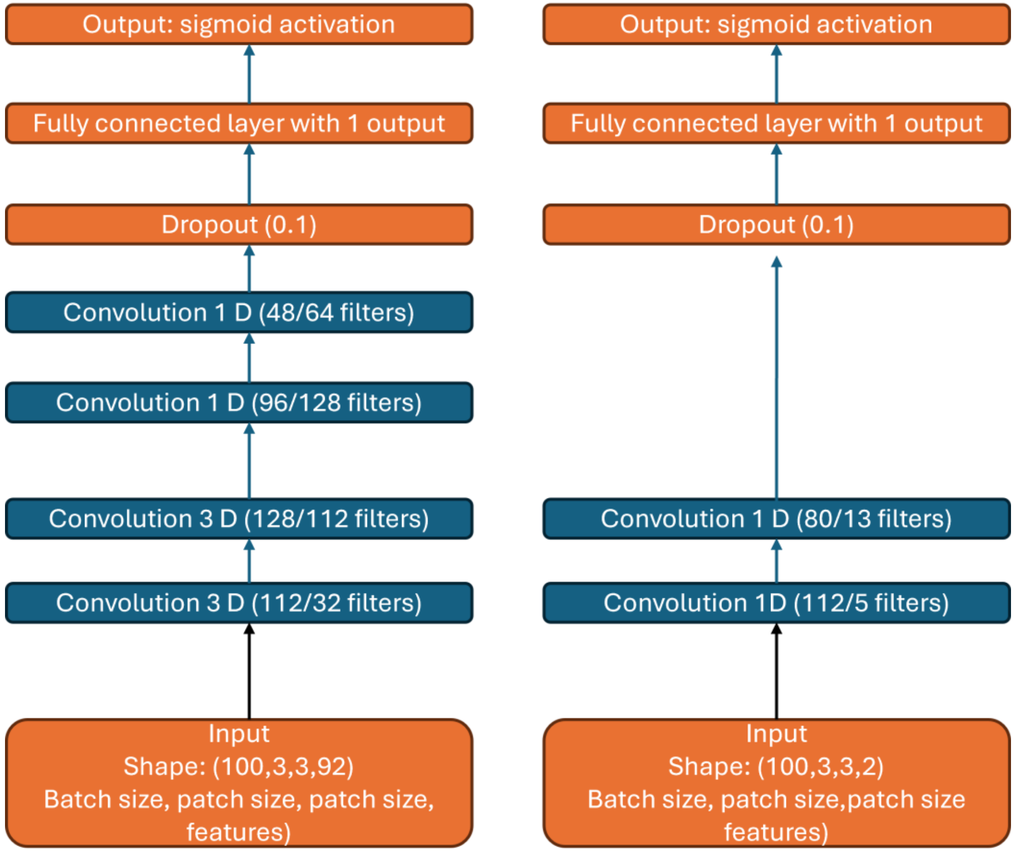

Supplementary Figure 1 *The Figure illustrates the Convolutional Neural Network architecture we used for classifying flap perfusion status based on hyperspectral imaging data. The figure presents two configurations: one tailored for spectral data (left) and another optimized for physiological parameters (right), both applied to datasets collected either on a single postoperative day or over multiple days. The CNN was designed with a series of 3D convolutional layers, followed by 1D convolutional layers, enabling hierarchical feature extraction from the hyperspectral data to enhance classification accuracy. To account for varying data distributions, filter sizes were adjusted dynamically depending on whether single-day or multi-day postoperative data were used.*
