# Supplementary material for: Detection of flap malperfusion after microsurgical tissue reconstruction using hyperspectral imaging and machine learning
Source: Sci Rep. 2025 May 5;15:15637. doi: 10.1038/s41598-025-98874-4 (PMC12052805; doi:10.1038/s41598-025-98874-4)
Supplement: Supplementary file 2 — Supplementary Information 2. [file 41598_2025_98874_MOESM2_ESM.pdf]

Supplementary materials

Table 1: Best hyperparameters and test results for using physiological parameters (pp), spectral data (sd)and standard machine learning methods (best results are shown bold).

method	input data	days	F1- Score (test results)	best parameters
Random Forest	sd	1	91,56	bootstrap: True, max_depth: 8, max_features: sqrt, n_estimators: 200
	sd	1 till 3	90,23	Bootstrap: True, max_depth: 8, max_features: sqrt, n_estimators: 200
	pp	1	88	Bootstrap: False, max_depth: 1, max_features: sqrt, n_estimators: 100
	pp	1 till 3	87,13	Bootstrap: True, max_depth: 8, max_features: sqrt, n_estimators: 100
Support Vector Machine	sd	1	99,61	C: 100, degree: 3, gamma: 1
	Sd	1 till 3	96,9	C: 1, degree: 4, gamma: 1
	Pp	1	82,6	C: 100, degree: 8, gamma: 1
	Pp	1 till 3	79,51	C: 100, degree: 1, gamma: 1
Multilayer Perceptron	sd	1	97,9	Activation: tanh, learning_rate: adaptive, solver: Adam
	sd	1 till 3	97,74	Activation: tanh, learning_rate: adaptive, solver: Adam
	pp	1	88,5	Activation: relu, learning_rate: constant, solver: Adam
	pp	1 till 3	87,75	Activation: relu, learning_rate: constant, solver: Adam
Logistic Regression	sd	1	84,04	C: 2.2, penalty: l1, solver: liblinear
	sd	1 till 3	87,02	C: 0.4, penalty: l1, solver: saga
	pp	1	88,31	C: 0.0001, penalty, l2, solver: liblinear
	pp	1 till 3	84,95	C: 0.001, penalty: l2, solver: liblinear
Convolutional Neural Network	**sd**	**1**	**100**	See supplementary figure 1, learning rate: 0.0005
	**sd**	**1 till 3**	**100**	See supplementary figure 1, learning rate: 0.0008
	**pp**	**1**	**89,7**	See supplementary figure 1, learning rate:0.002
	**pp**	**1 till 3**	**91.81**	See supplementary figure 1, learning rate:0.007
